# Women’s family-career conflict and its implications for job performance in the South African public sector: a systematic literature review

**DOI:** 10.3389/fsoc.2026.1865166

**Published:** 2026-10-09

**Authors:** Mosehlane Johannes Madingwaneng

**Affiliations:** 1 Royal Docks School of Business and Law, University of East London, London, United Kingdom; 2 Department of Sociology and Anthropology, University of Limpopo, Polokwane, South Africa

**Keywords:** employee performance, family-career balance, family-work conflict, family-career conflict, job performance, performance management, work-family conflict

## Abstract

**Introduction:**

Women’s family–career conflict (WFCC) has received growing attention, yet evidence on its implications for women’s job performance remains fragmented, particularly in the South African public sector. This study systematically reviews existing literature to examine the factors associated with WFCC, its implications for job performance, the role of organisational support and workplace policies, and the contextual conditions shaping these relationships.

**Methods:**

A systematic literature review was conducted using secondary scholarly literature, with the study-selection and reporting process informed by PRISMA 2020 guidelines. The literature was analysed thematically.

**Results:**

The findings show that WFCC does not have a uniform effect on job performance. While some studies associate higher levels of conflict with reduced performance and employee engagement, others report weak, mixed or context-dependent relationships. The review further shows that organisational support, flexible working arrangements, supportive leadership and family-friendly policies can reduce the pressures associated with competing family and career responsibilities. However, their effectiveness depends on organisational culture and how policies are implemented.

**Discussion:**

The study highlights the need for South African public-sector organisations to strengthen supportive workplace and performance-management practises that recognise employees’ family responsibilities.

## 1 Introduction

Employee job performance is central to the effectiveness of public organisations because it affects the quality, efficiency and reliability of services provided to citizens. Public institutions therefore rely on performance targets, accountability systems and performance management practises to ensure that employees meet expected standards. In South Africa, the importance of performance and accountability in the public service is well established, with government institutions expected to improve efficiency, service delivery and institutional performance (Department of Public Service and Administration, 2024). However, employee performance cannot be understood only in terms of organisational structures, management systems and performance targets. Employees also have responsibilities outside the workplace, and these responsibilities can influence how they experience and respond to the demands of their jobs.

This issue is particularly important for women. Over the past several decades, women’s participation in paid employment has increased across many parts of the world. However, this increase has not been accompanied by an equal sharing of unpaid household and care responsibilities. Women continue to carry a large proportion of childcare, household work and care for family members. The International Labour Organization ILO (2024) estimates that 708 million women worldwide are outside the labour force because of unpaid care responsibilities. These figures demonstrate that care remains strongly connected to women’s participation in the labour market. For women who are employed, the challenge is often not choosing between work and family, but managing the demands of both at the same time. The difficulty of managing these responsibilities has been widely examined through the concept of work–family conflict (WFC) and family–work conflict (FWC). WFC occurs when work demands interfere with family responsibilities, while FWC occurs when family responsibilities interfere with work responsibilities (Greenhaus and Beutell, 1985; Byron, 2005). The two forms of conflict are related but are not necessarily the same. A demanding work schedule, for example, may reduce the time available for family responsibilities and therefore create WFC. In contrast, childcare or other family responsibilities may make it difficult for an employee to meet work requirements, resulting in FWC. This distinction is important because the present study is particularly concerned with the family-to-work direction of conflict and how it may affect women’s ability to meet career and job expectations.

Research on WFC and FWC has grown considerably, with studies linking these forms of conflict to employee wellbeing, job satisfaction, burnout, engagement and other work-related outcomes (Byron, 2005; Cavagnis et al., 2023; Kim et al., 2024). However, the relationship between work–family conflict and performance is less straightforward. Some studies report that conflict can reduce performance because employees have limited time, energy and emotional resources available for their work roles. Other studies find weak or inconsistent relationships, suggesting that employees can sometimes maintain their performance despite experiencing conflict (Fan et al., 2024). This suggests that conflict does not automatically result in poor performance. Instead, its consequences may depend on the conditions under which employees work and the resources available to them.

The African context makes this issue particularly important. Work and family responsibilities are shaped not only by individual choices but also by social expectations, family structures, economic conditions and gender roles. In many African societies, women continue to be expected to perform substantial caregiving and household responsibilities while also participating in paid employment. These expectations can create competing demands between family and professional roles. However, much of the established WFC and FWC literature has developed in Western countries and private-sector settings. Findings from these contexts cannot always be transferred directly to African public-sector organisations because social expectations, employment conditions and institutional arrangements differ across contexts. Recent research also shows that cultural and organisational differences remain important in understanding how WFC develops and how employees respond to it (Sohal et al., 2025).

South Africa provides an important context for examining this relationship. Women form a substantial part of the country’s formal workforce, including the public service. At the same time, South African women continue to experience unequal responsibilities for unpaid domestic and care work. This creates a situation in which women may be expected to perform demanding professional roles while continuing to meet strong family and household expectations. The problem is therefore not simply the presence of family responsibilities. It is the interaction between these responsibilities and the conditions under which women are expected to perform their jobs.

The South African public sector is particularly relevant because employee performance is closely linked to service delivery, accountability and public confidence. Recent government and oversight reports continue to identify performance and accountability as important challenges for public institutions (Public Service Commission, 2025). Existing explanations for public-sector performance problems often focus on governance, leadership, corruption, institutional capacity and management systems (Mello, 2015; Fourie and Poggenpoel, 2017; Mokhatla et al., 2025). These explanations are important, but they tend to give less attention to the circumstances of individual employees. Understanding employee performance therefore requires consideration of both organisational conditions and the social realities that employees bring into the workplace. It is within this context that Women’s Family–Career Conflict (WFCC) becomes important. In this study, WFCC refers to the tension experienced by women when family responsibilities interfere with their professional responsibilities, career expectations and ability to manage competing roles. WFCC is closely related to FWC because both involve family demands affecting work. However, WFCC is used in this study to place greater emphasis on women’s experience of these competing family and career roles. This distinction allows the study to consider not only the occurrence of conflict but also the gendered conditions in which the conflict takes place.

An important gap in the existing literature is that much research on WFC and FWC has concentrated on psychological outcomes such as stress, burnout, wellbeing and job satisfaction. Less attention has been given to the implications of family-related conflict for measurable aspects of job performance, including productivity, efficiency, task completion and quality of work. This gap is important in the public sector because employee performance is not only a personal matter; it has consequences for organisational functioning and service delivery. At the same time, existing evidence does not support the simple assumption that WFCC necessarily causes poor job performance. A 2024 study, for example, found that WFC does not always reduce performance and showed that the relationship can depend on the level of support available to employees (Fan et al., 2024). This supports the need for a more careful and context-sensitive examination of the relationship. Organisational support is particularly important in this regard. Flexible working arrangements, supportive supervisors, family-friendly policies and understanding workplace cultures can help employees manage competing responsibilities. Recent meta-analytic evidence shows that family-friendly policies and workplace support can have positive effects on job and work–family outcomes, although their value can differ according to organisational and national context (Blom et al., 2025). Similarly, research on family-supportive supervisor behaviour indicates that supervisors can influence both work–family outcomes and employee performance (Ererdi et al., 2024). This suggests that the implications of WFCC may be shaped by the organisational environment in which women work rather than by family responsibilities alone.

As such, this study critically reviews the literature on WFCC and its implications for job performance in the South African public sector. The study is guided by Role Theory, which provides a useful basis for understanding the competing expectations attached to women’s family and professional roles. Rather than treating WFCC as a direct and universal cause of poor performance, the study considers how the relationship is shaped by social expectations, organisational conditions and available support. This approach allows the study to examine performance as something that occurs within a particular social and organisational context. The study has four objectives. First, it examines the socio-cultural, individual and organisational factors associated with WFCC. Second, it analysis existing evidence on the implications of WFCC for women’s job performance. Third, it examines the role of organisational support and workplace policies in shaping the relationship between WFCC and job performance. Fourth, it develops a context-sensitive interpretation of the WFCC–job performance relationship within the South African public sector. These objectives provide the direction for the review and ensure that the literature considered in the study remains focused on the relationship between women’s family responsibilities, career demands and job performance.

## 2 Significance of the study

### 2.1 Theoretical contribution

This study contributes to existing literature in two main ways. First, it moves beyond the dominant focus on psychological outcomes of WFC (Wang and Peng, 2017; Kim et al., 2024) and brings attention to performance-related implications. By linking gendered family responsibilities to measurable job performance outcomes (Triansyah et al., 2023), the study integrates gender studies with public sector performance discourse.

Second, it provides context-specific insight into the South African public sector. Much existing literature is developed in Western or private-sector contexts, limiting its relevance. By focusing on public institutions in South Africa, this study strengthens contextual understanding of how WFCC operates within bureaucratic systems.

### 2.2 Practical contribution

From a practical perspective, the study challenges the assumption that improving performance depends solely on stricter monitoring or structural reforms (Mello, 2015). If Women’s Family–Career Conflict has important implications for job performance, then gender-sensitive workplace strategies become necessary.

## 3 Literature review

### 3.1 Theoretical foundation: role theory

Role Theory provides the theoretical foundation for understanding Women’s Family–Career Conflict (WFCC) and its implications for job performance. The theory proposes that individuals occupy multiple social roles, with each role carrying particular expectations, responsibilities and behavioural obligations (Eagly and Wood, 2012; Anglin et al., 2022). Women may simultaneously occupy the roles of employee, mother, spouse, caregiver and community member, with each role requiring time, energy and emotional resources. Conflict may arise when the expectations associated with these roles become incompatible. Within this study, WFCC refers to tension that occurs when family responsibilities interfere with the fulfilment of career responsibilities. Job performance refers to the extent to which employees effectively fulfil their professional responsibilities, including task, contextual and adaptive performance (Borman and Motowidlo, 1993; Pradhan and Jena, 2017). Role Theory therefore provides a basis for examining how competing family and career expectations may influence the way women allocate time, energy and emotional resources across domains.

Importantly, Role Theory does not suggest that competing roles automatically result in poor performance. Rather, role conflict occurs when the demands associated with one role constrain the ability to fulfil another role. The consequences may therefore vary according to the intensity of competing demands and the resources available to individuals (Anglin et al., 2022). This distinction is important because the present study does not assume a direct or inevitable relationship between WFCC and poor job performance. The theory also recognises that role expectations are socially constructed. Gender norms influence expectations regarding caregiving, family responsibilities and professional commitment (Eagly and Wood, 2012). Women may consequently experience simultaneous expectations to perform effectively in the workplace while also assuming substantial responsibility for domestic and caregiving activities. WFCC must therefore be understood not only as an individual experience but also as a phenomenon shaped by social and organisational conditions.

### 3.2 Drivers of women’s family–career conflict

WFCC is rooted in the broader literature on work–family and family–work conflict, which describes incompatibility between demands arising from family and work domains (De Sousa, 2013; Raihen et al., 2023). Although conflict can occur in both directions, the experience of women requires particular attention because family responsibilities remain influenced by gendered expectations. Women continue to experience disproportionate responsibility for unpaid domestic and caregiving activities, which can create competing expectations between family and career roles (Eagly and Wood, 2012). The literature identifies both individual and structural explanations for WFCC. From an individual perspective, women may make choices concerning family involvement, career advancement and the allocation of time based on personal values and priorities (Madingwaneng et al., 2024; Garach, 2024). This perspective is important because it recognises women’s agency and their ability to negotiate competing responsibilities. However, interpreting WFCC primarily as a matter of individual choice risks overlooking the social conditions within which such choices are made.

A contrasting perspective emphasises socio-cultural and structural conditions. Gendered expectations may position caregiving and domestic responsibilities as primarily women’s responsibilities, while professional environments may continue to reward continuous availability and career commitment (Eagly and Wood, 2012). WFCC may therefore emerge when socially constructed family expectations conflict with organisational expectations. The conflict is consequently not produced by individual decisions alone but through the interaction between personal circumstances and the environments in which women perform their roles.

Organisational structures may further intensify these competing demands. Rigid working arrangements, limited flexibility and expectations of continuous workplace availability can make it more difficult for employees with substantial caregiving responsibilities to manage family and career roles. In contrast, organisational flexibility can provide women with greater capacity to negotiate competing demands (Soomro et al., 2018). Thus, the drivers of WFCC extend beyond individual characteristics to include organisational arrangements and wider socio-cultural expectations. The literature consequently suggests that WFCC is best understood as the outcome of interacting individual, socio-cultural and organisational factors. Focusing exclusively on either personal choices or structural constraints provides an incomplete explanation. Women exercise agency, but that agency operates within gendered family expectations, organisational policies and institutional conditions. This interaction provides a basis for examining how different conditions shape the subsequent implications of WFCC for job performance.

### 3.3 Women’s family–career conflict and job performance

The relationship between WFCC and job performance remains contested. Much of the existing literature associates family–career conflict with negative employee outcomes, including stress, emotional exhaustion, reduced job satisfaction and diminished organisational engagement (Wang and Peng, 2017; Kim et al., 2024). These outcomes suggest that competing family and career demands can consume cognitive and emotional resources that employees require to perform their professional roles effectively. Evidence specifically linking family–career conflict to job performance, however, is less consistent. Some studies report negative relationships between conflict and performance. Karatepe and Sökmen (2006), for example, associate conflict with reduced performance, while Sanz-Vergel et al. (2025) identify lower engagement in contexts characterised by family-related pressures. Andoh (2023) similarly reports negative implications associated with family interference. These findings support the argument that sustained conflict may limit the resources available for fulfilling professional responsibilities.

Other studies provide a less conclusive picture. Bhuian et al. (2005) found a weak or inconclusive relationship between family–work conflict and performance, while Netemeyer et al. (1996) reported no strong relationship between conflict and certain work outcomes. These findings are important because they challenge the assumption that experiencing WFCC necessarily results in reduced job performance. The contradictory findings indicate that the relationship between WFCC and performance may not be linear. Women may experience considerable family–career pressures while continuing to meet formal work requirements. The implications of conflict may depend on how individuals manage competing demands and, importantly, on the conditions within which they work. Organisational support, leadership, workplace culture and flexibility may influence whether family pressures are translated into observable performance difficulties.

The multidimensional nature of job performance further supports this interpretation. Performance extends beyond the completion of formal tasks to include contextual behaviours such as cooperation and organisational citizenship, as well as adaptive behaviours associated with responding to changing work requirements (Borman and Motowidlo, 1993; Pradhan and Jena, 2017). Family–career pressures may therefore affect different dimensions of performance differently. For example, an employee may continue meeting formal performance targets while experiencing difficulties with additional contextual or adaptive demands. The literature therefore does not justify viewing WFCC as an automatic explanation for women’s poor performance. Rather, the evidence suggests that WFCC may create conditions that have different implications depending on the organisational and social context. This distinction is particularly important in the South African public sector, where formal performance requirements coexist with employees’ family responsibilities.

### 3.4 Organisational support and workplace policies

The inconsistent relationship between WFCC and job performance draws attention to the role of organisational conditions. Organisational support and workplace policies can influence the extent to which women are able to manage competing family and career expectations. Flexible working arrangements, supportive leadership and family-friendly policies can provide employees with greater capacity to respond to family responsibilities without necessarily compromising professional obligations (Soomro et al., 2018).

Workplace flexibility is particularly relevant because the intensity of WFCC is partly determined by the degree to which employees can negotiate when and how work is performed. Rigid schedules may increase tension where family responsibilities require flexibility, whereas supportive arrangements may reduce the incompatibility between the two domains. Similarly, supportive leaders may assist employees in managing competing demands by creating an environment in which legitimate family responsibilities can be accommodated. The literature therefore suggests that organisational support should not be treated simply as an additional organisational benefit. It may influence how family–career pressures are experienced and how those pressures translate into workplace outcomes. Where organisational systems provide flexibility and support, women may be better positioned to maintain performance despite competing responsibilities. Conversely, rigid organisational structures may intensify existing pressures and make the consequences of WFCC more visible in workplace behaviour.

This perspective also challenges approaches that locate WFCC primarily within individual coping strategies. While individual coping remains relevant, organisational arrangements determine the resources and opportunities available for managing competing roles. The relationship between WFCC and job performance should therefore be examined in relation to the organisational environment in which women work. The literature further indicates that workplace policies alone may not be sufficient. The implementation of policies is shaped by leadership practises and organisational culture. A formal policy supporting flexibility may have limited value where employees perceive that using such arrangements signals weak commitment to their careers. Organisational support must therefore be considered in terms of both formal policies and the workplace conditions through which those policies are implemented.

### 3.5 Contextualising WFCC in the South African public sector

The South African public sector provides an important context for examining WFCC because employee performance occurs within formal institutional and accountability structures. Public-sector employees operate within systems characterised by performance requirements, formal hierarchies, compliance obligations and organisational accountability. Performance is commonly assessed through mechanisms such as Key Performance Indicators and other formal performance-management arrangements. Existing explanations of public-sector performance challenges in South Africa have largely focused on issues such as governance, leadership, corruption and organisational effectiveness (Mello, 2015; Fourie and Poggenpoel, 2017). Although these factors are important, such explanations may provide only a partial understanding of employee performance because they give limited attention to employees’ social and family circumstances. For women, formal workplace expectations may intersect with family responsibilities that require caregiving, emotional labour and physical presence within the household. Where organisational systems place strong emphasis on fixed schedules, continuous availability and formal performance targets, these expectations may create additional pressure for women managing substantial family responsibilities. The issue is therefore not whether women are capable of meeting performance expectations, but whether organisational arrangements recognise the social conditions within which those expectations must be fulfilled.

The relevance of context is reinforced by the geographical and sectoral concentration of existing research. Much of the literature on family–career conflict has been developed in Western and private-sector settings, where organisational practices, cultural expectations and employment conditions may differ from those experienced in the South African public sector. Applying findings from these settings directly to South Africa may therefore obscure important contextual differences. The South African context is particularly significant because gendered expectations concerning caregiving interact with formal institutional requirements. Where family responsibilities remain strongly associated with women, organisational structures that provide limited flexibility may intensify role conflict. Conversely, supportive organisational arrangements may allow women to manage competing expectations without necessarily experiencing substantial reductions in performance.

As such, the literature suggests that WFCC should not be conceptualised as a fixed individual-level problem with uniform consequences for job performance. Instead, its implications appear to depend on the interaction between individual circumstances, socio-cultural expectations and organisational conditions. This interpretation provides a more appropriate basis for understanding the inconsistent findings reported in previous research. The literature therefore points to a contextual understanding of WFCC in which organisational support, workplace policies, gendered expectations and institutional structures shape how competing family and career roles are experienced and managed. This perspective shifts the analytical question from whether WFCC affects job performance to the more useful question of under what conditions and through which organisational and social processes WFCC has implications for performance. Such a perspective is particularly relevant to the South African public sector, where formal performance systems operate alongside employees’ diverse family responsibilities.

## 4 Research methodology

### 4.1 Research approach and design

This study adopted a systematic literature review to analyse existing scholarship on WFCC and its implications for job performance in the South African public sector. This style of review provides a structured approach for identifying, screening, assessing and synthesising existing research in relation to clearly defined research objectives (Snyder, 2019; Page et al., 2021). The approach was appropriate because the study relied on secondary scholarly literature rather than primary data (Daniel et al., 2024; Sreekumar, 2023). The review was guided by four objectives: to examine the drivers of WFCC; to assess its implications for job performance; to examine the role of organisational support and workplace policies; and to extrnd Knowledge on context-sensitive interpretation of the WFCC–job performance relationship within the South African public sector. The reporting and study-selection process was informed by the Preferred Reporting Items for Systematic Reviews and Meta-Analyses (PRISMA) 2020 guidelines (Page et al., 2021).

### 4.2 Search strategy and data sources

The review used a structured search of academic literature through major multidisciplinary databases, principally Scopus and Web of Science. These databases were selected because of their broad coverage of peer-reviewed research in management, organisational studies, psychology, sociology and related social-science fields. The search focused on literature published between 1996 and 2025. The search strategy was developed directly from the research objectives and combined terms relating to WFCC, work–family conflict, job performance and organisational conditions. Search terms included “women’s family–career conflict”, “family–career conflict”, “work–family conflict”, “family–work conflict”, “women”, “job performance”, “employee performance”, “organisational support”, “workplace flexibility”, “family-friendly policies”, “public sector” and “South Africa.” Boolean operators such as AND and OR were used to combine and refine search terms. The search prioritised peer-reviewed scholarly literature and academically credible sources relevant to the research objectives. The use of a structured database search was intended to increase transparency and reduce the possibility of selectively identifying evidence (Snyder, 2019; Page et al., 2021). Reference lists of relevant studies were also considered where they provided additional evidence directly related to the objectives. The search therefore allowed the review to capture both South African evidence and theoretically relevant international and comparative studies.

### 4.3 Inclusion and exclusion criteria

Predefined inclusion and exclusion criteria were applied throughout the screening process. Studies were included when they addressed work–family, family–work or family–career conflict; examined women’s competing family and career responsibilities; considered job or employee performance or closely related workplace outcomes; examined organisational support, workplace flexibility, leadership or family-friendly policies; or provided evidence relevant to public-sector and organisational contexts. South African literature was prioritised because of the study’s geographical focus, while theoretically relevant comparative studies from other countries were included where they contributed directly to understanding the research objectives. English-language scholarly literature was included.

Studies were excluded when they had no meaningful connection to family–career or work–family conflict, did not address a relevant workplace outcome, had no organisational relevance, or did not contribute to any of the four research objectives. Non-scholarly opinion pieces and sources lacking sufficient academic relevance were also excluded. Explicit eligibility criteria provided a consistent basis for determining which studies formed part of the evidence base (Page et al., 2021). As the review was conducted by a sole researcher, all records were screened by the author against the predefined criteria. The same criteria were applied at both the title/abstract and full-text stages, and screening decisions were recorded to improve consistency.

### 4.4 Identification and selection of studies

The study-selection process followed the main stages of PRISMA 2020: identification, screening, eligibility assessment and inclusion (Page et al., 2021). Records retrieved from the database search were first examined and duplicates were removed. The remaining records were screened by title and abstract. Potentially relevant studies were then subjected to full-text assessment. Studies that satisfied the eligibility criteria were retained for the final evidence base (Table 1).

**Table 1:** PRISMA study identification and selection process.

PRISMA stage	Number
Records identified through database searching	304
Duplicate records removed	42
Records screened	262
Records excluded after title/abstract screening	183
Full-text reports assessed	79
Full-text reports excluded	41
Sources included in final evidence base	38

The selection process resulted in 38 sources being retained for analysis. The final evidence base contained different forms of scholarly evidence rather than only quantitative empirical studies. This was appropriate for the objectives because the review sought to understand not only the relationship between WFCC and performance but also its theoretical foundations, organisational conditions, policy environment and contextual dimensions.

### 4.5 Data extraction and methodological classification

Data were extracted from each included source according to its purpose, methodological approach, participants or evidence base, principal variables or themes, and relevance to the four research objectives (Taherdoost, 2022). To avoid treating different forms of evidence as methodologically equivalent, the 38 sources were classified according to their dominant methodological approach (Table 2).

**Table 2:** Methodological classification of the 38 included sources.

Evidence category	Number	Main purpose
Quantitative empirical	17	Statistical examination of conflict, support and workplace outcomes
Qualitative empirical	4	In-depth or thematic examination of experiences and contextual issues
Mixed methods	2	Integration of qualitative and quantitative evidence
Review/meta-analysis	7	Synthesis of existing empirical literature
Conceptual/theoretical	5	Development or explanation of theoretical constructs
Policy/institutional/statistical	3	Public-sector, labour-market and institutional context
Total	38	

### 4.6 Quantitative empirical evidence

Quantitative studies were used primarily to examine relationships between family–work or work–family conflict and job-related outcomes. They provided evidence concerning performance, burnout, engagement, job satisfaction, organisational support and other measurable outcomes. The quantitative evidence was not treated as establishing a single universal effect because the studies differed in samples, contexts, measures and analytical approaches (Table 3).

**Table 3:** Quantitative empirical studies included in the review.

Study	Method/sample	Main relevance
Andoh (2023)	Quantitative descriptive survey; 103 career women; questionnaires and descriptive/inferential statistics	Family-to-work conflict and job performance
Bhuian et al. (2005)	Quantitative survey and statistical modelling of sales employees	Role stressors, WFC and job outcomes
Cai et al. (2018)	Quantitative questionnaire; 167 employees; mediation analysis	Employee conditions and workplace outcomes
De Sousa (2013)	Quantitative electronic questionnaire; 545 employees	Family–work conflict, job satisfaction and burnout
Ererdi et al. (2024)	Two quantitative multisource/weekly diary studies	Family-supportive supervisor behaviour and work/home outcomes
Fan et al. (2024)	Three-wave time-lagged study; 378 employees; response-surface analysis and objective performance data	WFC, family support and safety performance
Frone et al. (1997)	Quantitative study; 372 employed adults; structural equation modelling	Work–family interface
Greenhaus et al. (1989)	Quantitative matched sample of 119 men and 119 women in two-career couples	Sources of work–family conflict
Karatepe and Sökmen (2006)	Quantitative survey of frontline hotel employees; path analysis	Work/family roles and psychological and behavioural outcomes
Karatepe and Tekinkus (2006)	Quantitative survey; 363 frontline retail-bank employees; LISREL/path analysis	WFC, emotional exhaustion and job outcomes
Kim et al. (2024)	Quantitative exploratory study; 151 working mothers of colour	WFC, burnout, gender-role beliefs and support
Netemeyer et al. (1996)	Quantitative scale-development study using three samples	Measurement of WFC and FWC
Patel et al. (2006)	Quantitative survey; 100 working mothers in a South African retail organisation	Family–work conflict and job performance
Sanz-Vergel et al. (2025)	Quantitative diary/multilevel study; 145 working couples; 1,450 daily observations	WFC and spouse/job performance
Singh et al. (2018)	Quantitative time-lagged study; 245 women engineers	WFC, occupational commitment and organisational support
Soomro et al. (2018)	Quantitative study; 280 faculty members in public-sector universities; regression analysis	WLB, WFC, FWC and employee performance
Wang and Peng (2017)	Quantitative questionnaire; 443 professional women; mediation analysis	WFC, job satisfaction and related outcomes

These studies provided the principal empirical evidence for examining associations between conflict and workplace outcomes. Their methodological differences were considered during synthesis rather than treating all reported relationships as directly comparable.

### 4.7 Qualitative and mixed-methods evidence

Qualitative studies provided contextual evidence concerning how women experience competing family and career responsibilities and how organisational and social conditions shape these experiences (Islam and Aldaihani, 2022). This evidence was particularly relevant to the study’s emphasis on context rather than assuming that WFCC operates uniformly across individuals or organisations (Tables 4, 5).

**Table 4:** Qualitative empirical studies.

Study	Method/data	Main relevance
Fourie and Poggenpoel (2017)	Qualitative/documentary analysis of Auditor-General and Public Service Commission reports	Public-sector inefficiencies and institutional conditions
Garach (2024)	Semi-structured in-depth interviews with 10 young South African Hindu women; thematic analysis	Gender expectations and anticipated WFC
Madingwaneng et al. (2024)	Qualitative approach; semi-structured interviews with 40 participants, supplemented by closed-ended items	Gendered employment and family/social conditions
Mokhatla et al. (2025)	Qualitative literature review of 45 research articles	Institutional and socio-economic conditions affecting South African local government

**Table 5:** Mixed-methods studies.

Study	Method/data	Main relevance
Pradhan and Jena (2017)	Qualitative interviews with researchers/practitioners followed by quantitative empirical validation	Development and validation of a multidimensional employee-performance framework
Raihen et al. (2023)	Quantitative survey with univariate, bivariate and multivariate analysis combined with qualitative interviews	Women’s career challenges and job satisfaction

The inclusion of qualitative and mixed-methods evidence complemented the quantitative literature by providing explanations of the circumstances in which conflict is experienced and managed.

### 4.8 Review, conceptual, theoretical and institutional evidence

Review and meta-analytic studies were included because they provided higher-level synthesis of existing evidence and helped situate the empirical studies within the broader WFC literature (Table 6).

**Table 6:** Review and meta-analytic studies.

Study	Review approach	Main contribution
Blom et al. (2025)	Meta-analysis of 229 independent samples and more than 1,600 effect sizes	Family-friendly policies, workplace support, career, job and work–family outcomes
Byron (2005)	Meta-analysis of more than 60 studies	Antecedents of work–family conflict
Cavagnis et al. (2023)	PRISMA-based systematic review of 13 studies	Protective factors for work–family conflict and women’s wellbeing
Mello (2015)	Critical literature review	Performance management and development in the South African public service
Sohal et al. (2025)	Systematic literature review using SPAR-4-SLR, bibliometric analysis and TCCM	Development of the work–family conflict research agenda
Thusi et al. (2023)	Literature-based secondary review	Public-sector performance-management challenges and opportunities
Triansyah et al. (2023)	Systematic review of seven studies	Factors affecting employee performance

The conceptual and theoretical sources were used to establish the analytical foundations of the review rather than to provide direct empirical estimates (Table 7).

**Table 7:** Conceptual, theoretical, policy, institutional and statistical sources.

Study	Type	Main contribution
Anglin et al. (2022)	Integrative/theoretical review	Role-theory perspectives and applications in management research
Borman and Motowidlo (1993)	Conceptual framework	Task and contextual dimensions of job performance
Eagly and Wood (2012)	Theoretical contribution	Social Role Theory and gendered role expectations
Evans and Davis (2005)	Conceptual/empirical framework	High-performance work systems, internal social structure and organisational performance
Greenhaus and Beutell (1985)	Conceptual literature model	Sources and dimensions of work–family role conflict
		
Department of Public Service and Administration (2024)	Government annual report	Public-service performance and institutional context
International Labour Organization ILO (2024)	Labour-market statistical report	Care responsibilities and women’s labour-force participation
Public Service Commission (2025)	Government annual report	Public-sector performance and accountability context

### 4.9 Theoretical and analytical lens

Role Theory was used as the theoretical and analytical lens for interpreting the reviewed literature. Role Theory explains how individuals occupy multiple roles carrying different expectations and responsibilities and how tensions may arise when the demands associated with these roles compete (Biddle, 1986; Eagly and Wood, 2012). In this study, the theory provides a basis for understanding how women negotiate family and career responsibilities and how the conditions surrounding these roles may influence workplace outcomes. The theory was not applied on the assumption that competing roles necessarily produce poor job performance. Rather, it was used to interpret how the intensity of role demands, available resources, organisational support and socio-cultural expectations may shape both the experience and consequences of WFCC. This approach is consistent with the study’s objective of examining the conditions under which WFCC has implications for performance.

### 4.10 Data analysis and thematic synthesis

The included literature was analysed using thematic synthesis. Thematic synthesis enables findings from different studies to be systematically compared, organised and interpreted around recurring concepts and patterns (Thomas and Harden, 2008; Caulfield, 2019; Jnanathapaswi, 2021). The analysis involved reading the selected sources, identifying recurring concepts and findings, comparing similarities and differences, and grouping related evidence into broader themes. The synthesis was organised around the four research objectives: (1) drivers of WFCC; (2) implications of WFCC for job performance; (3) organisational support and workplace policies; and (4) contextual conditions shaping the WFCC–job performance relationship within the South African public sector.

The analysis distinguished between evidence that reported negative performance-related outcomes, evidence reporting weak or mixed relationships, and evidence indicating that organisational or contextual conditions may shape the relationship. Contradictory findings were retained rather than removed because differences across studies may indicate that the relationship between WFCC and job performance is conditional rather than universal. This approach allowed the review to move beyond simply counting findings and instead consider patterns, differences and possible explanations across contexts.

### 4.11 Methodological alignment with the research objectives

The methodology was structured around the four research objectives to maintain consistency between the research problem, literature search, selection criteria and thematic analysis. The first objective guided the identification of evidence concerning socio-cultural, individual and organisational drivers of WFCC. The second guided the analysis of evidence concerning its implications for job performance. The third directed attention to organisational support, workplace flexibility, leadership and family-friendly policies. The fourth guided interpretation of the institutional and contextual conditions relevant to the South African public sector. This alignment enabled the review to move beyond a general discussion of women, employment and family responsibilities and instead synthesise literature directly relevant to the relationship between WFCC and job performance.

### 4.12 Researcher consistency and transparency

As a single-researcher systematic review, consistency was maintained by applying the same predefined eligibility criteria throughout the screening process. Records were considered first at title and abstract level and then, where potentially relevant, at full-text level. The study-selection numbers were retained at each PRISMA stage to provide a transparent account of how the final evidence base was established. The methodological classification of the included sources also ensured that quantitative, qualitative, mixed-methods, review, conceptual and institutional evidence were distinguished during interpretation. This was important because the different evidence types answer different aspects of the research problem. Quantitative studies provided evidence of measurable relationships, qualitative studies contributed contextual and experiential insight, review studies provided broader analysis, while theoretical and institutional sources supported interpretation of the social and organisational environment in which WFCC occurs. The methodology was designed to provide a systematic and transparent interpretation of existing evidence while recognising differences in research design, geographical context, outcome measures and theoretical orientation. The approach therefore supports a context-sensitive interpretation of WFCC and its implications for women’s job performance rather than assuming a uniform causal relationship.

## 5 Findings and data presentation

### 5.1 Findings

#### 5.1.1 Drivers of women’s family–career conflict

The first objective examined the socio-cultural, individual and organisational factors associated with WFCC. Three broad categories emerged from the literature: socio-cultural expectations, organisational or structural conditions, and individual factors. The first finding concerns the influence of gendered social expectations. Women continue to experience expectations relating to caregiving, family nurturing and domestic responsibilities, while simultaneously being expected to meet workplace responsibilities. Social Role Theory suggests that such expectations are shaped by socially constructed understandings of gender and the roles associated with women and men (Eagly and Wood, 2012). The literature reviewed therefore indicates that WFCC cannot be understood solely as an individual difficulty because family and career responsibilities are embedded within wider social expectations.

The second category concerns organisational and structural conditions. Rigid working arrangements, limited flexibility and formal organisational structures may restrict women’s ability to respond to competing family and career demands. This issue is particularly relevant to public-sector organisations, where formal performance-management arrangements and institutional procedures may limit employees’ discretion over when and where work is performed (Mello, 2015; Thusi et al., 2023). The evidence therefore suggests that organisational arrangements can either intensify or reduce the tension experienced between family and career roles. The third category concerns individual circumstances and values. The reviewed literature indicates that women differ in how they prioritise and negotiate family and career responsibilities. Personal values and individual circumstances may therefore influence how competing roles are experienced (Madingwaneng et al., 2024). However, the findings do not support an interpretation in which WFCC is simply a consequence of individual choices. Individual preferences operate within broader socio-cultural and organisational conditions.

### 5.2 Implications of WFCC for job performance

The second objective examined existing evidence concerning the implications of WFCC for women’s job performance. The evidence was mixed and did not establish a consistent or universal relationship. Several studies reported negative workplace outcomes associated with work–family or family–career conflict. Karatepe and Tekinkus (2006) associated work–family conflict with adverse job outcomes, while Patel et al. (2006) identified a relationship between family–work conflict and job performance among working mothers. More recent evidence also indicates that work–family conflict may be associated with lower employee engagement and other adverse workplace outcomes (Sanz-Vergel et al., 2025). These findings suggest that sustained conflict between family and work responsibilities can coincide with reduced workplace functioning.

However, the evidence also contains findings that do not support a straightforward negative relationship. Bhuian et al. (2005) reported a more complex relationship between work-related stressors and job outcomes, while Netemeyer et al. (1996) demonstrated that work–family and family–work conflict are distinct constructs that should not automatically be treated as direct predictors of a single workplace outcome. The reviewed evidence therefore does not justify the conclusion that WFCC inevitably produces poor job performance. The findings further indicate that job performance should be understood as a multidimensional construct. Performance may involve task performance, contextual performance and adaptive performance, meaning that conflict may affect different dimensions of performance differently (Borman and Motowidlo, 1993; Pradhan and Jena, 2017). Consequently, an employee may continue to complete formal work tasks while experiencing difficulties in other aspects of workplace functioning.

### 5.3 Organisational support and workplace policies

The third objective examined the role of organisational support and workplace policies in shaping the experience of WFCC and its implications for job performance. The evidence indicates that workplace flexibility is an important organisational condition. Flexible working arrangements can provide employees with greater capacity to manage family and work responsibilities and have been associated with lower levels of work–family conflict and improved employee outcomes (Soomro et al., 2018). Supportive leadership also appears to be relevant because employees’ ability to manage competing responsibilities is influenced not only by formal organisational policies but also by how those policies are experienced in practise (Singh et al., 2018).

In contrast, rigid organisational systems may intensify role pressures. Evidence concerning public-sector performance-management systems indicates that bureaucratic procedures and institutional constraints can create challenges for effective performance management (Thusi et al., 2023). Mello (2015) similarly highlights the importance of organisational structures and performance-management arrangements within the South African public service.

The findings therefore suggest that the existence of a family-friendly policy does not necessarily mean that employees experience effective organisational support. The practical value of such policies depends on their implementation, organisational culture and the degree of flexibility available to employees. Organisational support should consequently be understood as a condition that can shape how women experience and manage competing family and career responsibilities rather than as a guaranteed solution to WFCC.

### 5.4 Contextual conditions shaping WFCC in the South African public sector

The fourth objective sought to develop a context-sensitive interpretation of the WFCC–job performance relationship within the South African public sector. The findings indicate that the relationship should be interpreted within the institutional conditions in which women perform their jobs. South African public-sector organisations operate through formal accountability, performance-management and administrative structures. Existing research on public-sector performance has largely focused on organisational and institutional factors, including performance-management systems, governance and administrative capacity (Mello, 2015; Mokhatla et al., 2025; Thusi et al., 2023). The findings from this review indicate that employee-level family responsibilities should also be considered when understanding workplace performance. The evidence does not, however, establish that South African public-sector women experience WFCC in exactly the same way. Differences in organisational support, job characteristics, family circumstances and institutional flexibility may produce different experiences and outcomes. The reviewed evidence therefore supports a contextual interpretation rather than a universal causal claim. A further finding is that the relationship between WFCC and job performance appears to depend partly on the conditions surrounding the employee. Where employees have access to flexibility, supportive leadership and appropriate organisational resources, family and career demands may be more manageable (Singh et al., 2018; Soomro et al., 2018). Conversely, rigid organisational arrangements may intensify competing role demands (Mello, 2015; Thusi et al., 2023).

### 5.5 Overall findings

The four objectives collectively produce one central finding: WFCC should not be treated as a universal or direct determinant of women’s job performance. Instead, its implications appear to vary according to the interaction between individual circumstances, gendered social expectations, organisational structures and workplace support (Table 8).

**Table 8:** The overall findings of the study.

Research objective	Main finding	Key supporting literature
Drivers of WFCC	WFCC is shaped by socio-cultural expectations, organisational structures and individual circumstances.	Eagly and Wood (2012), Madingwaneng et al. (2024), Mello (2015), Sohal et al. (2025), Sanz-Vergel et al. (2025), Patel et al. (2006), Anglin et al. (2022)
WFCC and job performance	The relationship is inconsistent, with evidence of negative, weak and non-significant outcomes.	Bhuian et al. (2005), Karatepe and Sökmen, 2006, Karatepe and Tekinkus (2006), Patel et al. (2006), Sanz-Vergel et al. (2025)
Organisational support	Flexibility and supportive organisational practises can reduce the difficulty of managing competing roles, while rigid systems may intensify conflict.	Singh et al. (2018), Soomro et al. (2018), Thusi et al. (2023)
South African public-sector context	The implications of WFCC need to be interpreted in relation to institutional and organisational conditions rather than assumed to be universal.	Mello (2015), Mokhatla et al. (2025), Thusi et al. (2023)

The overall findings therefore supports the study’s central proposition that WFCC is context-dependent. The evidence does not demonstrate that family–career conflict invariably results in poor job performance. Instead, the implications appear to depend on how family demands interact with social expectations, organisational structures and the resources available to women to manage competing roles.

## 6 Discussion

### 6.1 Interpreting WFCC as a context-dependent phenomenon

The findings suggest that WFCC should not be understood as having a uniform relationship with job performance. The implications of competing family and career responsibilities appear to vary according to the social expectations placed on women, the organisational environment in which they work, and the extent to which workplace arrangements accommodate competing responsibilities. From a Role Theory perspective, these differences reflect the way individuals negotiate multiple and sometimes competing role expectations across work and family domains (Eagly and Wood, 2012; Anglin et al., 2022). The findings further indicate that the relationship between WFCC and job performance is shaped by organisational conditions. Where women have access to supportive leadership, workplace flexibility and organisational resources, competing family and career demands may be more manageable. In contrast, rigid working arrangements, limited flexibility and unsupportive organisational practises may intensify role pressures and make it more difficult for women to sustain expected levels of job performance.

The study contributes a context-sensitive analysis of existing evidence, showing that the implications of WFCC for job performance vary according to social, organisational and institutional conditions. Rather than suggesting that WFCC has a uniform relationship with job performance, the review indicates that organisational support, workplace flexibility, leadership practises and broader social expectations can shape how women experience and respond to competing family and career demands. This interpretation also helps explain the inconsistent findings reported in the literature. Differences in organisational settings, forms of support, leadership practises and measures of job performance may contribute to variation in how WFCC is associated with workplace outcomes. Thus, the evidence reviewed does not support a simple linear explanation in which greater WFCC necessarily results in poorer job performance. Instead, the relationship appears to be conditional on the circumstances in which women negotiate their family and career roles.

### 6.2 Understanding the inconsistent WFCC–job performance relationship

One of the most important findings of the review is the absence of a consistent relationship between WFCC and job performance. Some studies associate family-career conflict with reduced performance, lower engagement and weaker organisational commitment (Karatepe and Sökmen, 2006; Karatepe and Tekinkus, 2006; Patel et al., 2006; Sanz-Vergel et al., 2025). Other studies report weak or non-significant relationships (Bhuian et al., 2005; Netemeyer et al., 1996). The significance of this variation is that WFCC should not be presented as an inevitable cause of poor performance. One possible explanation is that job performance itself has several dimensions. Performance includes the completion of core work responsibilities but can also involve contextual and adaptive behaviours (Borman and Motowidlo, 1993; Pradhan and Jena, 2017). Family-career pressures may therefore affect some aspects of work behaviour more strongly than others. An employee may continue to complete formal duties while experiencing difficulties with concentration, participation, cooperation or adaptation. Consequently, studies using different measures of performance may arrive at different conclusions.

The inconsistency may also reflect differences in organisational circumstances. Employees do not experience family-career conflict in the same institutional environment. Leadership practices, workplace culture, flexibility and available resources can influence whether competing responsibilities become visible in work outcomes. The findings therefore suggest that the relationship should be interpreted as conditional rather than linear. This interpretation is particularly important for public-sector performance debates. Poor performance should not automatically be attributed to individual employees without considering the conditions under which performance is expected. At the same time, the review does not suggest that WFCC explains public-sector performance problems generally. Rather, it identifies family-career demands as one workforce-related factor that may interact with wider organisational and institutional conditions.

### 6.3 Organisational support as a condition shaping outcomes

The literature reviewed indicates that organisational support can influence how women manage competing family and career demands. Flexible working arrangements, supportive leadership and family-friendly practises may provide employees with greater capacity to manage responsibilities across the two domains (Singh et al., 2018; Soomro et al., 2018). The significance of these practises is therefore not simply that they reduce the existence of family responsibilities, but that they can change the conditions under which those responsibilities are managed.

However, the review also indicates that the existence of a policy does not necessarily guarantee its effectiveness. Formal organisational arrangements may have limited value where implementation is constrained by rigid procedures, bureaucratic requirements or limited organisational capacity (Thusi et al., 2023). This distinction between policy availability and policy implementation is important in understanding WFCC in the public sector. The implication is that organisational support should be considered as part of the environment within which role conflict occurs. Where employees have reasonable flexibility and supportive supervisors, competing demands may be more manageable. Where organisational arrangements are rigid, the same family responsibilities may create greater pressure. Thus, organisational support does not necessarily eliminate WFCC; rather, it can shape the extent to which conflict translates into difficulties at work.

### 6.4 Implications for the South African public sector

The South African public-sector context gives particular importance to these findings because employee performance occurs within formal systems of accountability, performance management and service delivery. Existing discussions of public-sector performance commonly emphasise governance, management, accountability and institutional weaknesses (Mello, 2015; Mokhatla et al., 2025). The present review suggests that workforce experiences should also form part of this broader discussion.

This does not mean that WFCC should replace established explanations of public-sector performance problems. Instead, it adds another dimension to the analysis. Women working within public institutions may be expected to meet formal performance requirements while simultaneously managing family and caregiving responsibilities. Where organisational arrangements provide limited flexibility, these competing expectations may become more difficult to manage. The relevance of this observation is therefore institutional rather than gender-essentialist. The issue is not that women are inherently more likely to perform poorly, but that organisational systems may fail to recognise the social conditions within which employees perform their duties. A performance system that evaluates outputs without considering the organisational conditions surrounding employees may provide an incomplete understanding of performance.

### 6.5 Theoretical implications

The review contributes to Role Theory by demonstrating the value of considering context when examining competing roles. Role Theory explains why incompatible expectations can generate role conflict, but the findings suggest that the consequences of this conflict cannot be understood independently of the environment in which roles are performed (Eagly and Wood, 2012; Anglin et al., 2022). The contribution of the present review is therefore not to propose that family-career conflict always reduces performance. Instead, it suggests that role conflict should be understood as a process whose consequences can vary according to organisational and institutional conditions. This interpretation helps explain why previous studies have produced different results. The review consequently advances a context-based conceptualisation of WFCC. Under this view, WFCC represents an interaction between competing role expectations and the conditions available for managing those expectations. This provides a stronger basis for interpreting mixed evidence than a model that assumes a direct and universal negative relationship between WFCC and performance.

### 6.6 Practical implications

The findings have implications for human resource management and performance management in the public sector. First, organisations should consider whether performance systems provide sufficient flexibility for employees managing legitimate family responsibilities. This does not require lowering performance standards. Rather, it requires considering whether the conditions under which employees are expected to achieve those standards are supportive and realistic. Second, organisational support should extend beyond the formal adoption of family-friendly policies. Implementation, supervisory attitudes and workplace culture are important because employees may not benefit from policies that are difficult to access or inconsistently applied. Supportive leadership can therefore form an important part of creating an environment in which employees can manage competing responsibilities. Third, performance management should avoid treating all variations in employee performance as evidence of individual failure. The review suggests that organisational conditions can influence how competing roles are experienced and managed. A more responsive approach would therefore combine accountability with appropriate organisational support. These implications should nevertheless be interpreted cautiously. Because the present study is based on existing literature, it does not provide empirical evidence that a particular intervention will directly improve performance. Instead, it identifies organisational support and workplace conditions as areas that deserve greater attention in future policy and management practise.

### 6.7 Contribution of the study

The main contribution of this review is its integration of three areas that are often examined separately: women’s family-career responsibilities, employee job performance and South African public-sector organisational conditions. The review shows that examining these areas together provides a more nuanced explanation of the inconsistent evidence surrounding WFCC. The study also contributes by challenging a purely individualised interpretation of family-career conflict. The findings indicate that women’s experiences are influenced not only by personal choices and family responsibilities but also by the organisational conditions within which professional roles are performed. This provides a basis for understanding WFCC as a context-dependent phenomenon rather than as a fixed individual problem. Overall, the review suggests that the most useful question is not simply whether WFCC affects job performance, but under what organisational and institutional conditions family-career conflict is more or less likely to have performance-related consequences.

## 7 Conclusion

This study examined the implications of WFCC for job performance within the South African public sector through a systematic review of existing literature. The review demonstrates that the relationship between WFCC and job performance cannot be understood as a simple or universal cause-and-effect relationship. Instead, its implications vary according to the social, organisational and institutional conditions in which women perform their professional and family roles. The study contributes a context-specific synthesis of existing evidence, showing that the implications of WFCC vary according to social, organisational and institutional conditions. Role Theory provides a useful explanation of how competing expectations associated with family and professional roles can create tension, but the consequences of this tension are influenced by the environment in which those roles are managed. Organisational support, workplace flexibility, leadership practises and performance-management arrangements can therefore shape how family-career pressures are experienced and whether they translate into work-related difficulties. For the South African public sector, this finding has particular significance. Public-sector performance is generally understood through accountability, targets, compliance and service-delivery requirements. However, these systems operate through employees whose work and family responsibilities are interconnected. The review therefore suggests that efforts to improve performance should not focus exclusively on monitoring and accountability, but should also consider the organisational conditions that enable employees to perform effectively. This does not imply reducing performance standards; rather, it calls for performance systems that recognise the realities of the workforce. The study also highlights the importance of moving beyond the existence of family-friendly policies to their effective implementation. Flexible work arrangements, supportive supervision and appropriate organisational practises may assist women in managing competing responsibilities. However, their effectiveness depends on organisational culture, managerial practises and institutional capacity. Public-sector organisations should therefore consider how existing human resource and performance-management arrangements can better accommodate legitimate family-care responsibilities while maintaining accountability. Despite these contributions, the study is limited by its reliance on secondary literature. The available evidence is also drawn from different organisational and national contexts, which limits the extent to which conclusions can be generalised specifically to South African public institutions. The review should therefore be understood as providing a conceptual and analytical foundation rather than definitive empirical evidence of a causal relationship between WFCC and job performance. Future research should address this limitation through empirical studies involving women employees across different levels and departments of the South African public sector. Quantitative studies could examine the strength and direction of relationships between WFCC, organisational support and different dimensions of job performance, while qualitative research could provide deeper insight into how women experience and manage competing roles within public institutions. Comparative research across public-sector departments could also establish whether organisational culture, leadership and flexibility produce different outcomes. Overall, the study concludes that addressing WFCC is not only a matter of employee wellbeing but also a relevant consideration in human resource and performance management. A more context-sensitive approach to family and career responsibilities may contribute to healthier workplaces while supporting effective and sustainable public-sector performance.

## Data Availability

The original contributions presented in the study are included in the article/supplementary material, further inquiries can be directed to the corresponding author.
